# The impact of endometrial thickness change after progesterone administration on pregnancy outcome in patients transferred with single frozen-thawed blastocyst

**DOI:** 10.1186/s12958-019-0545-0

**Published:** 2019-11-25

**Authors:** Zhiqin Bu, Xinhong Yang, Lin Song, Beijia Kang, Yingpu Sun

**Affiliations:** Reproductive Medical Center, Henan Province Key Laboratory for Reproduction and Genetics, The First Affiliated Hospital of Zhengzhou University, 1# Jianshe East, Zhengzhou, Henan Province China

**Keywords:** Infertility, Endometrium, IVF/ICSI outcome

## Abstract

**Background:**

The aim of this study was to explore the impact of endometrial thickness change after progesterone administration on pregnancy outcome in patients transferred with single frozen-thawed blastocyst.

**Methods:**

This observational cohort study included a total of 3091 patients undergoing their first frozen-thawed embryo transfer (FET) cycles between April 2015 to March 2019. Endometrial thickness was measured by trans-vaginal ultrasound twice for each patient: on day of progesterone administration, and on day of embryo transfer. The change of endometrial thickness was recorded.

**Results:**

Regardless of endometrial preparation protocol (estrogen-progesterone/natural cycle), female age, body mass index (BMI), and infertility diagnosis were comparable between patients with an increasing endometrium on day of embryo transfer and those without. However, clinical pregnancy rate increases with increasing ratio of endometrial thickness. Compared with patients with Non-increase endometrium, those with an increasing endometrium on day of embryo transfer resulted in significantly higher clinical pregnancy rate (56.21% vs 47.13%, *P* = 0.00 in estrogen-progesterone cycle; 55.15% vs 49.55%, *P* = 0.00 in natural cycle).

**Conclusions:**

In most patients, endometrial thickness on day of embryo transfer (after progesterone administration) increased or kept being stable compared with that on day of progesterone administration. An increased endometrium after progesterone administration was associated with better pregnancy outcome.

## Introduction

For decades, the debate about the relationship between endometrial thickness and clinical pregnancy has never stopped [1, 2]. At present, most studies have observed that thin endometrium has an adverse effect on pregnancy outcome during in vitro fertilization (IVF) treatment, even though the specific mechanism is not well understood [3–5].

Why scholars are keen to study the relationship between endometrium and pregnancy outcome? One of the important reasons is that endometrial thickness may represent endometrial receptivity [6]. Under physiological conditions, endometrial receptivity mainly refers to the ability of the endometrium to accept embryos when implantation window is open, which is around 7 days after ovulation in natural menstrual cycle. In patients undergoing IVF treatment, the day of embryo transfer is usually considered to be in the middle of implantation window. Thus, it is reasonable to believe that the condition of endometrium on day of transfer, but not on day of human chorionic gonadotropin (hCG) trigger or progesterone administration (both are at least 3–5 days before embryo transfer), is more representative of endometrial receptivity.

It is known that the condition of endometrium is changeable in natural menstrual cycle, and also in IVF treatment. One of the typical change is that endometrial pattern will be changed from pattern A (triple-line pattern)/pattern B (intermediate isoechogenic pattern) to pattern C (homogenous, hyperechogenic pattern) after hCG or progesterone administration during IVF cycles [7, 8], but little is known about the endometrial thickness change after hCG or progesterone administration. In the morning of embryo transfer, endometrial pattern and thickness of all patients are routinely re-evaluated by trans-vaginal ultrasound in our center. Thus it is convenient for us to record the change of endometrial thickness after progesterone administration in frozen-thawed embryo transfer (FET) cycles.

The aim of the current study was to record the dynamic change of endometrial thickness (Day of embryo transfer Versus. Day of progesterone administration), and to investigate the impact of endometrial thickness change on pregnancy outcomes in women with single blastocyst transfer in their first FET cycles.

## Materials and methods

In 2015 we registered this observational cohort study on Clinical Trial (NCT02420704). In total, 3091 patients undergoing their first FET cycles [1757 Estrogen-progesterone (EP) cycles and 1334 Natural cycles (NC)] from April 2015 to March 2019 were included. Data in this study were from the Clinical Reproductive Medicine Management System/Electronic Medical Record Cohort Database (CCRM/EMRCD) in Reproductive Medical Center, First Affiliated Hospital of Zhengzhou University. This study has been approved by The Institutional Review Board of Zhengzhou University.

Only patients with single blastocyst transfer were included. Endometrial pattern was pattern A/B on day of progesterone administration, and was pattern C on day of embryo transfer for all patients.

For exclusion criteria, patients with low quality embryo (a blastocyst score < 3 BC according to Gardner system) or with thin endometrial thickness (< 7 mm on day of progesterone administration) were also excluded. In addition, pre-implantation genetic diagnosis cycles, as well as oocyte donation cycles were also not included.

Preparation of endometrium for FET was EP and NC. The detailed protocol for endometrial preparation, endometrial pattern classification, and also the method of how to measure endometrial thickness, all have been described in our previous work [3].

### Endometrial thickness on day of progesterone administration

For EP cycles, only oral estradiol ([progynova]; Bayer, Germany) were used in the first 12–14 days. Once endometrial thickness reached ≥7 mm, progesterone in oil (60 mg) was added. For NC cycles, progesterone in oil (40 mg) was first added on the day of ovulation. Endometrial thickness on this progesterone administration day will be recorded by trans-vaginal ultrasound examination.

### Endometrial thickness on day of embryo transfer

All FET patients in our center were hospitalized the day before embryo transfer as a routine practice. Endometrial thickness was re-evaluated on the morning of embryo transfer day, also by trans-vaginal ultrasound, to exclude cavity fluid and other unfavorable conditions.

After embryo transfer, luteal supplement was changed to vaginal progesterone gel (90 mg, Crinone 8%; Merck Serono) and oral dydrogesterone (20 mg duphaston; Abbott). Clinical pregnancy was confirmed by ultrasound observation 5 weeks after embryo transfer.

### Statistical analysis

Patient with endometrial thickness on day of embryo transfer thicker than that on day of progesterone administration were allocated into the Increasing Group. The Non-increase Group included patients with endometrial thickness on day of embryo transfer equal to, or less than that on day of progesterone administration.

Student’s t-test was used to detect difference between continuous variables, and chi-square test were for categorical variables. Statistical analysis was performed with SPSS (Statistical Package for Social Science, SPSS Inc., Chicago, IL, USA) version 19.0. A *P* < 0.05 was considered to be statistically significant.

## Results

**Fig. 1:**
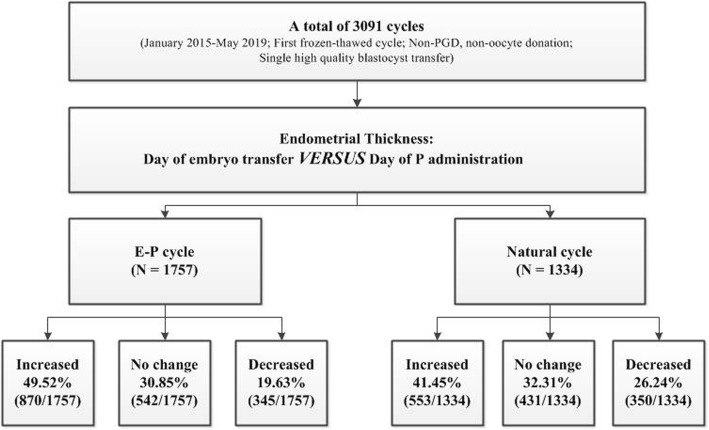
Study flow chart. *PGD* preimplantation genetic diagnosis, *P* progesterone, *E-P* estrogen-progesterone

In this observational cohort study, a total of 3091 first FET cycles with single blastocyst transfer were included. A brief description and flow chart of this study was shown in Fig. 1. In EP cycle, endometrial thickness on day of embryo transfer decreased in 19.63% patients, and this proportion was even higher (26.24%) in NC cycle.

Table 1 shows the basic character and pregnancy outcomes in patients according to the change of endometrial thickness. Regardless of EP or NC cycle, female age, body mass index (BMI), and infertility diagnosis were comparable between those with an increasing endometrium on day of embryo transfer and those without. However, for both EP and NC cycles, endometrial thickness on day of progesterone administration in Increasing Group was significantly thinner at first (9.03 ± 1.34 vs 9.61 ± 1.57, *P* = 0.00 in EP cycle; 9.64 ± 1.70 vs 10.69 ± 1.96, *P* = 0.00 in NC cycle); it increased dramatically at the end and became thicker on day of embryo transfer when compared with that in Non-increase Group (10.60 ± 1.66 vs 9.11 ± 1.49, *P* = 0.00 in EP cycle; 11.25 ± 1.93 vs 10.03 ± 1.82, *P* = 0.00 in NC cycle).

**Table 1:** Patient basic characteristic and pregnancy outcomes in women transferred with single frozen thawed blastocyst

	Estrogen-progesterone cycle (*N* = 1757)			Natural cycle (*N* = 1334)		
	Increasing (*N* = 870)	Non-increase (*N* = 887)	*P*	Increasing (*N* = 553)	Non-increase (*N* = 781)	*P*
Age (years)	30.27 ± 4.57	30.63 ± 4.98	0.11	31.31 ± 4.64	31.76 ± 4.96	0.10
Body Mass Index (Kg/m^2^)	22.67 ± 3.22	22.73 ± 3.27	0.71	22.64 ± 3.23	22.65 ± 3.19	0.97
History of prior gravidity (%)	55.17% (480/870)	56.37% (500/887)	0.61	67.09% (371/553)	63.38% (495/781)	0.16
Infertility diagnosis						
Tubal factor	36.32% (316/870)	36.30% (322/887)		51.72% (286/553)	52.75% (412/781)	
Ovulation disorder	26.78% (233/870)	27.96% (248/887)	0.75	−	−	0.94
Male factor	21.49% (187/870)	18.49% (164/887)		3.15% (128/553)	23.30% (182/781)	
Combined and others	15.40% (134/870)	17.25% (153/887)		25.14% (139/553)	23.94% (187/781)	
Endometrial thickness: starting progesterone	9.03 ± 1.34	9.61 ± 1.57	0.00	9.64 ± 1.70	10.69 ± 1.96	0.00
Endometrial thickness: embryo transfer	10.60 ± 1.66	9.11 ± 1.49	0.00	11.25 ± 1.93	10.03 ± 1.82	0.00
Clinical pregnancy rate (%)	56.21% (489/870)	47.13% (418/887)	0.00	55.15% (305/553)	49.55% (387/781)	0.04
Ectopic pregnancy rate (%)	1.43% (7/489)	2.39% (10/418)	0.29	1.64% (5/305)	1.55% (6/387)	0.93
Early spontaneous abortion rate (%)	14.1% (69/489)	18.66% (78/418)	0.06	12.77% (40/305)	13.70% (53/387)	0.82

**Fig. 2:**
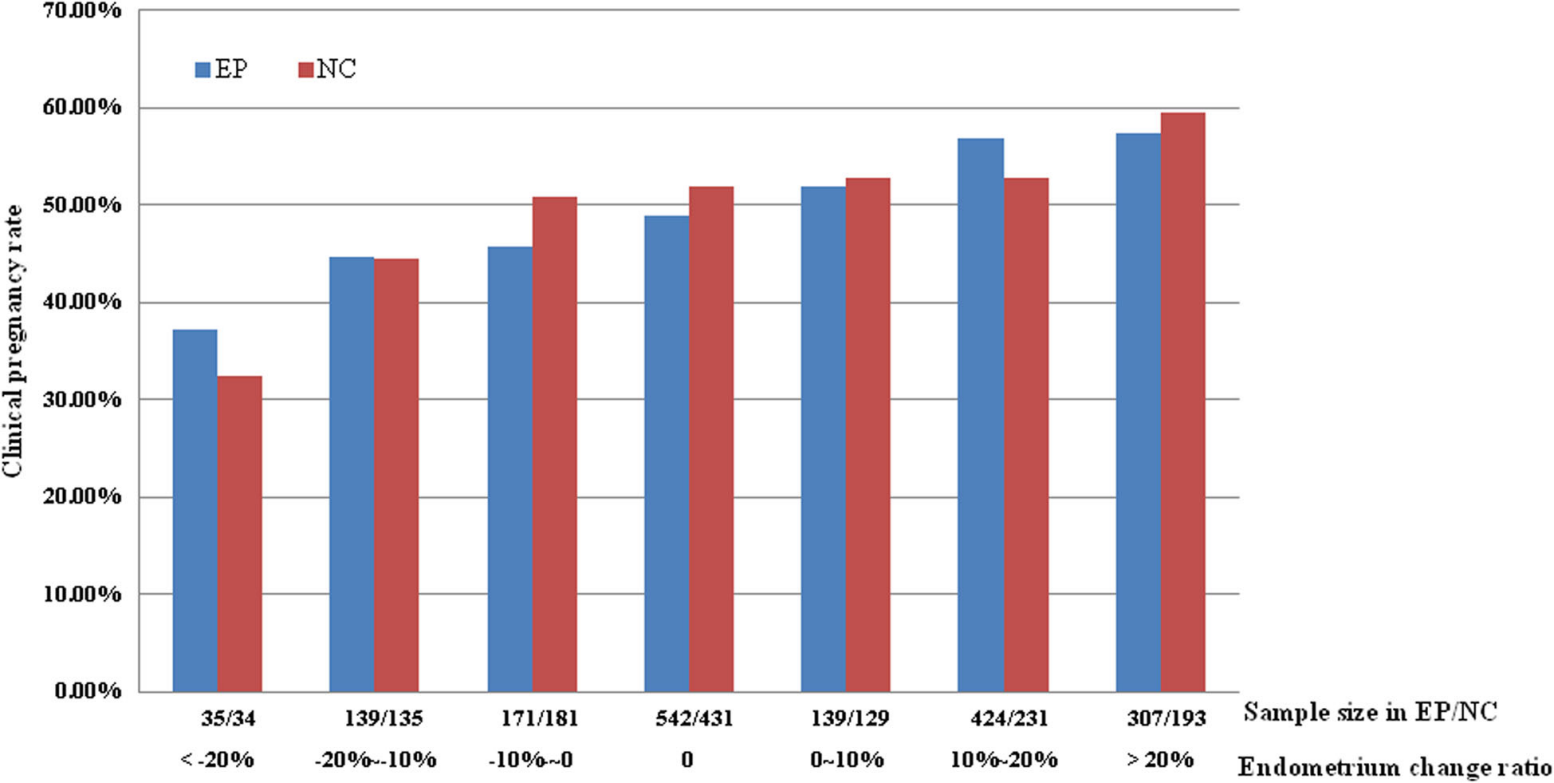
Relationship between endometrium change ratio and clinical pregnancy rate. *EP* estrogen-progesterone, *NC* natural cycle

As for the pregnancy outcome, Fig. 2 clearly shows that clinical pregnancy rate increases with increasing ratio of endometrial thickness. Even though with limited participants, in endometrial thickness decreasing ≥20% group, clinical pregnancy rate in EP and NC cycles was only 37.14 and 32.35%, respectively. However, in endometrial thickness increasing ≥20% group, clinical pregnancy rate was nearly 60% in both EP and NC cycles. In addition, Table 1 also shows that for patients either in EP or NC cycle group, compared with patients with Non-increase endometrium, those with an increasing endometrium on day of embryo transfer resulted in significantly higher clinical pregnancy rate (56.21% vs 47.13%, *P* = 0.00 in EP cycle; 55.15% vs 49.55%, *P* = 0.00 in NC cycle). Moreover, early spontaneous abortion rate in Non-increase group was slightly higher even without statistical significant. Ectopic pregnancy rate was also comparable in Increasing group and Non-increase group irrespective of EP or NC cycles.

## Discussion

To date, numerous studies, including our previous work, have focused on the impact of endometrial thickness on IVF outcome, and the conclusions are basically the same: a thin endometrium (< 7 mm in most cases) is predicable for a poor pregnancy outcome [9, 10]. Recently, a cohort study including over 40,000 participants from Canada, and another UK study with over 20,000 cycles both showed similar results [11, 12].

In most previous studies, endometrial thickness was measured during ovarian stimulation (in fresh embryo transfer cycles), or during proliferation phase before progesterone administration (in frozen thawed embryo transfer cycles), both are several days ahead of embryo transfer. However, in 2009 Barker et al have noticed this issue and evaluated the relationship between endometrial thickness in luteal phase (day of ET), in the late follicular phase, and pregnancy outcome in oocyte donation model [13]. Data from this study showed that endometrial thickness in recipients prior to and following progesterone had no impact on pregnancy outcomes. More recently, another cohort study including 271 FET cycles also evaluated endometrial thickness on both the end of the estrogen phase and the day of embryo transfer [14]. Interestingly, they found that a decreased endometrium (endometrial compaction) in response to progesterone results in better pregnancy outcome in FET cycles, which means that the greater the degree of compaction, the higher the ongoing pregnancy rate. In Barker’s study, endometrial thickness in pregnant and non-pregnant group after progesterone administration increased by 1.3 and 0.7 mm, respectively. Our study also showed that in 3091 cycles, endometrial thickness on day of embryo transfer increased by 0.41 mm on average compared with the day of progesterone administration(from 9.73 mm to 10.14 mm). In addition, the ratio of patients with endometrial compaction > 5% was 21.96% (679/3091) in our study, however, this is far less than that (42.44% = 115/271) from Haas’s study.

Question is, how does the endometrial thickness change during the proliferation phase and the luteal phase under physiological conditions? In natural menstrual cycle, estrogen and progesterone secreted by the corpus luteum makes the endometrium from the proliferative phase continue to grow. Endometrial gland is more curved, and secretion occurs. Meanwhile, blood vessels rapidly increased, and endometrial stroma became loose. It was shown that endometrial thickness increase during the follicular phase of the menstrual cycle, peak prior to ovulation, plateau during the early luteal phase and then decline prior to menstruation [15]. Thus, an increased endometrium after progesterone administration in FET cycles seemed to be a reasonable phenomenon, and we speculated that pregnancy was much easier in patients with an increased endometrium, which is consistent with results from our study.

In addition, during FET procedure, estrogen level may differ between NC cycles and EP cycles after progesterone administration. In EP cycles, oral estradiol is continued after progesterone administration; however, in NC cycles, only progesterone was used after ovulation without any estrogen supplementation. It is reasonable to find that endometrial thickness increased 0.52 mm on average in EP cycles, but it only increased 0.28 mm on average in NC cycles from our study.

Even though as an observational cohort study, our large sample size study indeed has several strengths. First, only high quality, single blastocyst transfer cycles were included, because number of embryo and quality of embryo are very important factors related with pregnancy outcome in IVF treatment cycles. Second, patients with thin endometrium (< 7 mm) was excluded as situation of this group of patients was complicated. In addition, we included patients in FET transfer with both EP and NC cycles. Patients in our center with single blastocyst transfer in fresh embryo transfer cycles are high ovarian responders. Ovarian stimulation protocols are different, and are out of control for further analysis. Another point is that the endometrial situation of each patients is re-evaluated on the morning of embryo transfer day also by trans-vaginal ultrasonic examination, which is a more accurate way to measure endometrial thickness. In Haas’s study, endometrial thickness was measured by means by pelvic (transabdominal) ultrasound [14]. This may explain the controversy between the totally opposite results from these two studies.

Table 2 briefly shows the endometrial thickness on day of progesterone, on day of embryo transfer, in patients with different endometrial thickness change group. It warn us endometrial thickness examination after progesterone administration is important. Patients with dramatic decrease in endometrial thickness do have good endometrium at first, but they do not result in final optimistic outcome.

**Table 2:** Endometrial thickness on day of progesterone administration/embryo transfer according endometrial change ratio

	Endometrial change ratio	< −20%	−20%~ − 10%	−10%-~ 0	0	0~10%	10%~ 20%	> 20%
EP cycle	No.	35	139	171	542	139	424	307
	Age (year)	30.77	30.61	30.68	30.61	30.55	30.3	30.11
	Thickness on dPA (mm)	10.8	9.66	10.22	9.33	9.96	8.92	8.77
	Thickness on dET (mm)	7.96	8.25	9.36	9.33	10.69	10.01	11.36
NC cycle	No.	34	135	181	431	129	231	193
	Age (year)	31.44	32.05	31.83	31.66	31.21	31.16	31.59
	Thickness on dPA (mm)	12.76	10.88	11.07	10.31	11.04	9.23	9.19
	Thickness on dET (mm)	9.28	9.17	10.14	10.31	11.91	10.38	11.84

*EP* estrogen-progesterone, *NC* natural cycle, *dPA* day of progesterone administration, *dET* day of embryo transfer; Endometrial change ratio = (Thickness on dET- Thickness on dPA)/ Thickness on dPA*100%

However, several limitations exist in this study. First, the specific molecular and cellular evident behind this phenomenon needs to be explored in future studies. In addition, as a preliminary observational study, no additional clinical management was performed if endometrium did not increase, because endometiral thickness was ≥7 mm in these cases. Moreover, even each physician in our center are well-trained, and are capable of ultrasonic examination under Standard Operation Procedure, inter-observer variability for endometrial thickness may bring some bias to this study.

## Conclusion

In summary, the current large sample size observational cohort study demonstrated the dynamic change of endometrial thickness after progesterone administration in FET cycles. In both EP and NC cycles for endometrium preparation, endometrial thickness on day of embryo transfer increased or kept being stable compared with that on day of progesterone administration in most patients. In addition, an increased endometrium after progesterone administration was associated with better pregnancy outcome. Interestingly, clinical pregnancy outcomes and the increasing rate of endometrium were positively correlated.

## Data Availability

All data supporting the conclusion of this article are included.

## Abbreviations

BMI: Body mass index
EP: Estrogen-progesterone
FET: Frozen thawed embryo transfer
hCG: Human chorionic gonadotropin
ICSI: Intracytoplasmic sperm injection
IVF: In ivtro fertilization
NC: Natural cycle
